# Arabidopsis Polycomb Repressive Complex 2 binding sites contain putative GAGA factor binding motifs within coding regions of genes

**DOI:** 10.1186/1471-2164-14-593

**Published:** 2013-08-30

**Authors:** Weiwei Deng, Diana M Buzas, Hua Ying, Masumi Robertson, Jennifer Taylor, William James Peacock, Elizabeth S Dennis, Chris Helliwell

**Affiliations:** 1 CSIRO Plant Industry, GPO Box 1600, Canberra ACT 2601, Australia; 2 Plant Reproductive Genetics, Graduate School of Biological Sciences, Nara Institute of Science and Technology, 8916-5 Takayama, Ikoma, Nara 630-0192, Japan

**Keywords:** Polycomb, Chromatin immunoprecipitation, H3K27me3

## Abstract

**Background:**

Polycomb Repressive Complex 2 (PRC2) is an essential regulator of gene expression that maintains genes in a repressed state by marking chromatin with trimethylated Histone H3 lysine 27 (H3K27me3). In Arabidopsis, loss of PRC2 function leads to pleiotropic effects on growth and development thought to be due to ectopic expression of seed and embryo-specific genes. While there is some understanding of the mechanisms by which specific genes are targeted by PRC2 in animal systems, it is still not clear how PRC2 is recruited to specific regions of plant genomes.

**Results:**

We used ChIP-seq to determine the genome-wide distribution of hemagglutinin (HA)-tagged FERTLIZATION INDEPENDENT ENDOSPERM (FIE-HA), the Extra Sex Combs homolog protein present in all Arabidopsis PRC2 complexes. We found that the FIE-HA binding sites co-locate with a subset of the H3K27me3 sites in the genome and that the associated genes were more likely to be de-repressed in mutants of PRC2 components. The FIE-HA binding sites are enriched for three sequence motifs including a putative GAGA factor binding site that is also found in Drosophila Polycomb Response Elements (PREs).

**Conclusions:**

Our results suggest that PRC2 binding sites in plant genomes share some sequence features with Drosophila PREs. However, unlike Drosophila PREs which are located in promoters and devoid of H3K27me3, Arabidopsis FIE binding sites tend to be in gene coding regions and co-localize with H3K27me3.

## Background

The Polycomb group (PcG) proteins are found across the higher eukaryotes and are essential for normal development. PcG proteins were first identified in Drosophila where they are required to maintain repression of homeotic genes [1] and have since been shown to be required for the correct expression of many genes in plants and animals. The polycomb proteins make up two major protein complexes; Polycomb Repressive Complex 1 (PRC1) and PRC2 [1-5] which are conserved in animals and plants. PRC2 catalyses trimethylation of histone H3 lysine 27 (H3K27me3). The H3K27me3 is bound by PRC1 which ubiquitinates histone H2A [4,6] resulting in a compacted chromatin state that can be inherited through mitotic divisions.

Plants have clear homologs of the four core protein components of PRC2, often with multiple genes encoding each component [7]. In Arabidopsis, FERTILIZATION INDEPENDENT ENDOSPERM (FIE) is the single Extra Sex Combs (ESC) homolog, CURLY LEAF (CLF), SWINGER (SWN) and MEDEA are Enhancer of Zeste homologs, FERTILIZATION INDEPENDENT SEED 2, VERNALIZATION 2 and EMBRYONIC FLOWER 2 are Suppressor of Zeste 12 homologs and MULTI-SUBUNIT SUPPRESSOR OF IRA (MSI) 1–5 are homologs of NURF55. Of the MSIs, MSI1 appears to be a component of PRC2 complexes, linking PRC2 to LIKE HETEROCHROMATIN PROTEIN 1 [8], a protein that has PRC1-like function in Arabidopsis, while other MSIs (e.g. MSI4/FVE) have roles outside the PRC2 complex [7,9]. A loss of PRC2 activity in Arabidopsis, such as in *clf swn* double mutants and *FIE* RNAi plants leads to strong developmental defects, especially in organ identity [10,11]. Whole genome chromatin immunoprecipitation (ChIP) experiments have shown that about 20% of Arabidopsis genes are marked by H3K27me3 [12-17]. The H3K27me3 targets have an over-representation of genes that are highly regulated as opposed to being constitutively expressed. H3K27me3 is generally associated with genes with low transcription activity [3,16] consistent with H3K27me3 having a role in maintaining repression of gene expression. Although large numbers of gene loci have H3K27me3 present, only a minority are de-repressed in vegetative tissues of plants mutant for PRC2 components [13]. This indicates that in these tissues H3K27me3 is only critical for maintaining the repression of a subset of the H3K27me3-marked genes and presumably loss of H3K27me3 at other loci does not lead to their increased expression due to the absence of specifically expressed transcription factors.

A major unanswered question in understanding Polycomb repression in plants is how specific loci are targeted by the Polycomb complexes. Polycomb recruitment has been best characterised in Drosophila where regions of H3K27me3 are associated with sequence elements termed Polycomb Response Elements (PREs) [18]. Drosophila PREs are regions of up to a few hundred base pairs that were initially defined as being required to confer Polycomb repression on their target genes. PREs are able to recruit either PRC1 or PRC2 or both. PREs contain binding sites for sequence-specific DNA binding proteins. The binding sites for Pleiohomeotic (Pho) and the related Pho-like are a common element of Drosophila PREs, but they also contain sites for other DNA binding proteins including GAGA factor, Pipsqueak and Zeste. Genome-wide studies show that the binding sites for these other factors only partially overlap with PRC1 and PRC2 target sites and the extent to which they contribute to PcG recruitment is not always clear [4,18].

In mammalian systems, PREs are less well characterised with the best examples being a 3 kb region from the mouse MafB gene and a 1.8 kb region from the human *HOXD* cluster that confer PcG-dependent repression in reporter gene systems [19,20]. Both these elements contain binding sites for YY1 (the mammalian PHO homolog) suggesting that there is at least some conservation of the mechanisms of PcG recruitment between mammals and insects. Long non-coding RNAs (lncRNAs) have also been implicated in PcG recruitment in mammals. These can act in *cis*, such as the Xist and Kcnq1ot1 lncRNAs that are involved in PcG recruitment in X chromosome inactivation and imprinting respectively [21,22], or in *trans*, such as the HOTAIR lncRNA which is produced from the *HOXC* cluster and acts as a scaffold to recruit PRC2 to the unlinked *HOXD* locus [23,24].

At present less is known of the mechanisms by which genes are targeted by the PcG system in plants. There is some evidence for the presence of PRE-like sequences in plants. A 50 bp element (RLE) has been identified from the promoter of the Arabidopsis *LEAFY COTYLEDON 2* (*LEC2*) gene which is required for PcG repression and confers repression and H3K27me3 deposition on a transgene [25]. The *LEC2* promoter also contains a GAGA element that is bound by Arabidopsis GAGA factors *in vitro*. Mutation studies suggest that this GAGA element has an activator or enhancer function and is not required for H3K27me3 deposition [25]. A second example of a plant PRE-like sequence comes from the promoter of the *BREVIPEDICELLUS* (*BP*) gene. The ASYMMETRIC LEAVES 1 (AS1)–AS2 complex binds to defined sequences in the *BP* promoter to silence its expression [26]. The *BP* locus is marked by H3K27me3 which requires the AS1-AS2 complex. The AS1-AS2 complex interacts with PRC2 components and the AS1-AS2 binding site from the *BP* promoter is sufficient to confer Polycomb repression on a GUS transgene. These properties are consistent with the AS1-AS2 binding site in *BP* functioning as a PRE at which AS1-AS2 recruits PRC2 [27].

Some evidence for lncRNAs being involved in Polycomb recruitment in plants comes from the *FLC* gene which encodes a MADS box repressor of flowering [28,29]. *FLC* expression is repressed by vernalisation (extended cold) and this repression is maintained in a PRC2-dependent manner following return to warm growing conditions [11,30]. Non-coding sense transcripts (named COLDAIR) produced from the large first intron of *FLC* which are bound by the PRC component CLF, are required to maintain *FLC* repression in the cold [31], suggesting that the COLDAIR transcript recruits PRC2 to maintain *FLC* repression.

To further explore the mechanisms of PcG recruitment in plants we carried out a ChIP-seq experiment to determine the genome-wide distribution of FIE, the single ESC homolog in Arabidopsis which should therefore be present in all PRC2 complexes. By comparing the FIE binding sites with genome-wide H3K27me3 distribution we found over seven hundred high confidence FIE binding sites. The FIE binding sites were predominantly within gene bodies and were enriched for three sequence motifs including putative GAGA factor binding sites.

## Results

### Identification of FIE-HA binding sites by ChIP-seq

**Figure 1:**
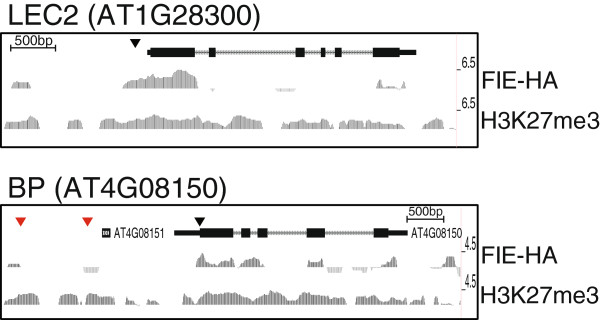
**FIE**-**HA and H3K27me3 distribution at the *LEC2 *and *BP *loci.** Broswer images showing the FIE-HA and H3K27me3 distribution across the *LEC2* and *BP* loci. Black arrows are locations of GAGA motifs identified in *LEC2*[25], red arrows are AS1-AS2 binding sites in the *BP* promoter [27].

The FIE protein is the only Arabidopsis homolog of the ESC protein and is present in all active PRC2 complexes. Hence ChIP using the FIE protein as a target should identify all sites of PRC2 interaction with the genome. We carried out ChIP-seq with a FIE-HA protein complementing the *fie*-*11* mutant in the C24 ecotype [11] using aerial tissue of 12 day old seedlings. As the PRC2 complex is thought to interact with either histones or DNA binding proteins and not directly with genomic DNA, we cross-linked the plant material prior to immunoprecipitation to preserve the interaction of FIE-HA with its chromatin binding sites. In a parallel experiment, ChIP-seq was also carried out for H3K27me3 using non cross-linked chromatin from wildtype C24 seedlings. This native ChIP method is generally considered superior for assessing histone modifications [32] while cross-linked ChIP is preferred for studying chromatin binding proteins. The ChIP-seq datasets were mapped to the Arabidopsis genome and regions that were enriched in the immunoprecipitations compared to input samples were identified as described in Methods. Figure 1 shows example data for the *LEC2* and *BP* genes. This identified 1298 peaks in the FIE-HA data and 5148 peaks in the H3K27me3 data using a q score of 10^-10^. This q score was selected from a range that was tested on the basis that it identified the largest number of peaks from the ChIP samples with minimal false positive peaks from the input datasets (Additional file 1: Table S1; Additional file 2: Dataset S1, Additional file 3: Dataset S2).

### H3K27me3 abundance and distribution is conserved between C24 and Columbia ecotypes

**Figure 2:**
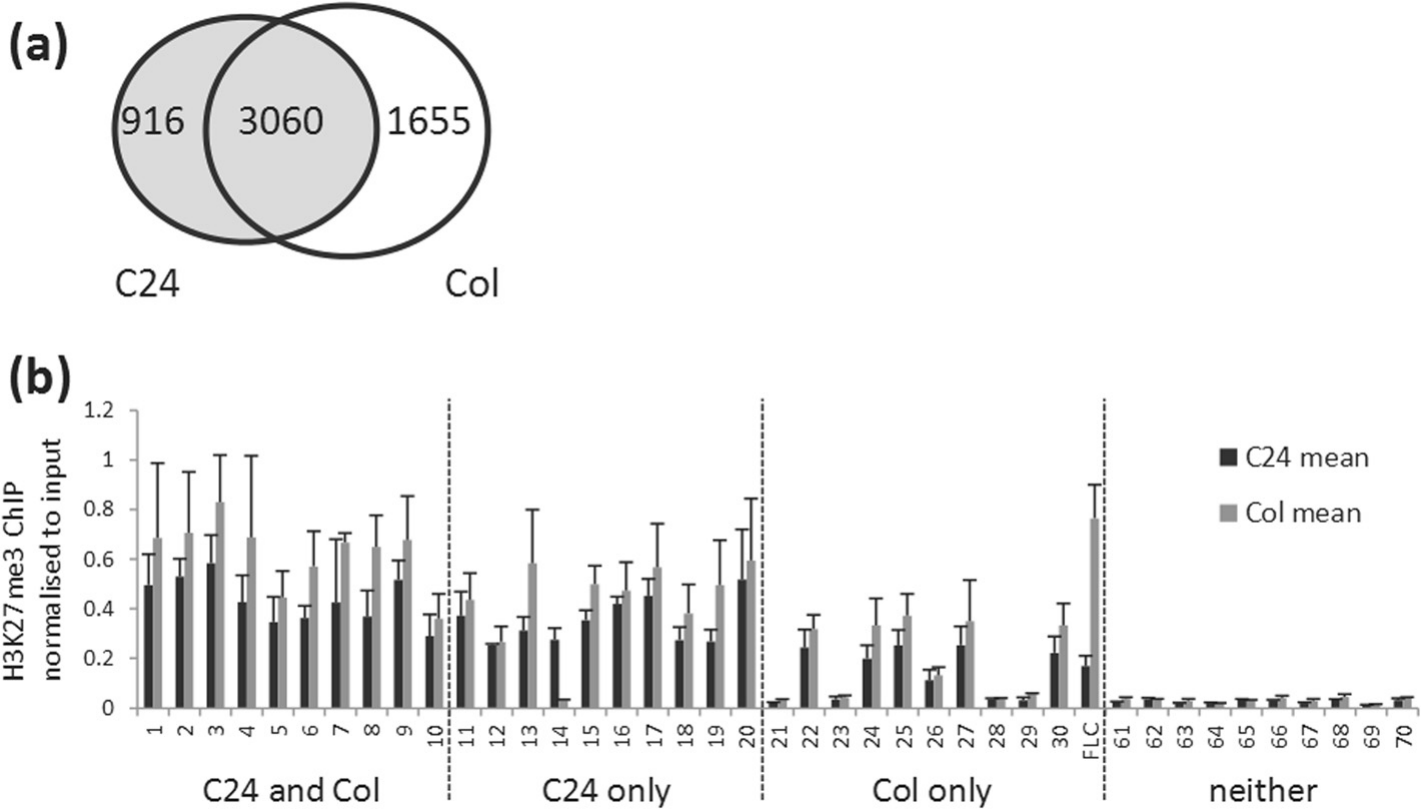
**H3K27me3 distribution is conserved between C24 and Col. (a)** Numbers of genes corresponding to an H3K27me3 ChIP-seq peak in C24 (this study) and Col [16] showing the overlap between the two datasets. **(b)** H3K27me3 ChIP-qPCR enrichment normalised to input DNA for amplicons from regions identified as enriched in C24 and Col (1–10), C24 only (11–20), Col only (21–30) and neither (61–70). An amplicon from *FLC* (Amplicon 5a) [35] is included for comparison.

The number of H3K27me3 peaks we identified was similar to that seen in previous analyses [16]. To determine whether these represent the same target sequences we compared the H3K27me3 distribution in our C24 dataset to a Columbia (Col) Chip-chip dataset [16]. The peaks identified in our C24 H3K27me3 dataset were assigned to 3976 genes, 77% of which were also present in the list of H3K27me3 genes from Col (Figure 2a). To verify this result and quantitatively compare the H3K27me3 abundance between the two ecotypes, 10 genes were randomly selected from each of three groups; present in C24+Col, C24-only and Col-only. We determined the H3K27me3 abundance at the centre of the peaks for these 30 genes, as well as for 10 regions with no H3K27me3, by ChIP-qPCR on Col and C24 seedlings grown under the same conditions (Figure 2b). We found that all 10 C24+Col genes had similar H3K27me3 abundance in both ecotypes. We found that 18 out of 20 genes selected as having either C24- or Col-specific H3K27me3 actually had similar H3K27me3 abundance suggesting that there are false negatives in both datasets. Therefore there are very few differences in H3K27me3 location and abundance between ecotypes, in agreement with previous observations for Col versus L*er*[33] and Col versus C24 or Cvi [34].

### FIE-HA peaks are associated with H3K27me3

**Figure 3:**
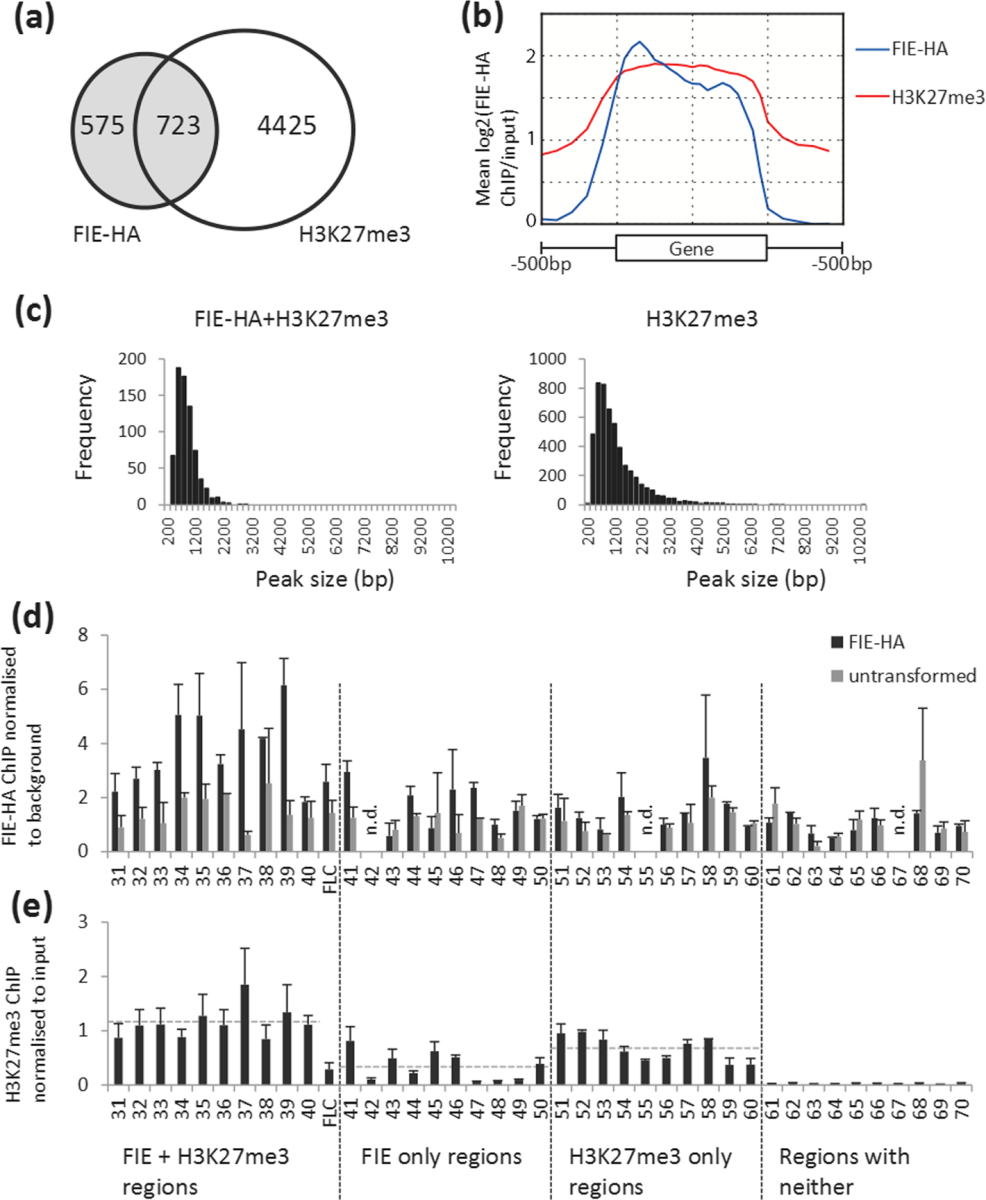
**FIE binding coincides with H3K27me3 peaks. (a)** Overlap of FIE-HA and H3K27me3 ChIP-seq peaks. **(b)** Distribution of FIE-HA and H3K27me3 ChIP-seq signal relative to gene coding regions. **(c)** Size distribution of FIE-HA+H3K27me3 and H3K27me3 peaks. Peaks were placed in 200 bp size bins. **(d)** Verification of FIE-HA ChIP-seq peaks by ChIP-qPCR. Amplicons correspond to peaks present in both FIE-HA+H3K27me3 (1–10), FIE-HA only (41–50), H3K27me3 only (51–60) and neither dataset (61–70); values are normalized to the average signal of the untransformed control. Amplicons 61–70 are as used in Figure 2. **(e)** H3K27me3 ChIP-qPCR for genes present in FIE-HA+H3K27me3, FIE-HA only, H3K27me3 only and neither dataset; values are enrichment relative to input DNA. (n.d.; not determined). Dotted lines are average H3K27me3 abundance for H3K27me3 + FIE (does not include *FLC* as this was not associated with an H3K27me3 peak), FIE-HA only and H3K27me3 only regions. Averages are 1.15, 0.34 and 0.67; the averages for H3K27me3+FIE and H3K27me3 only are different at p<0.001 and FIE-only and H3K27m3-only are different at p<0.01 using a two-tailed *t*-test.

The genomic locations of the peaks from our FIE-HA and H3K27me3 ChIP-seq datasets were compared and grouped into three categories; FIE-HA+H3K27me3, FIE-HA only and H3K27me3 only (Figure 3a; Additional file 4: Dataset S3). We found that 723 of the FIE-HA peaks overlapped with H3K27me3 peaks (an overlap of 1bp or more). To verify the ChIP-seq results we then selected two peaks at random from each of five confidence value bins for each category of ChIP-seq peak for further analysis by ChIP-qPCR (detailed in Methods). In addition to the 10 peaks for each of the three groups, we included the 10 regions with no H3K27me3 (amplicons 61–70), which were also not FIE-HA targets. The amplicons were placed in the centre of the overlap of the FIE-HA+H3K27me3 peaks or the centre of the FIE-HA or H3K27me3 only peaks. For ChIP-qPCR, new sample sets immunoprecipitated for FIE-HA and H3K27me3 were prepared. As a positive control we carried out qPCR with *FLC* amplicon 5a located in the first exon of *FLC* which has detectable FIE-HA binding and low but detectable H3K27me3 [35]. We found enrichment of H3K27me3 at all 20 H3K27me3 sites tested, confirming that the number of false positives in the H3K27me3 dataset is low (Figure 3e). Of the 10 FIE-HA only regions, five showed a higher enrichment of H3K27me3 above background than the *FLC* control primers, again showing that there are false negatives for H3K27me3 (Figure 3e). The regions selected as having neither H3K27me3 nor FIE-HA present had very low levels of H3K27me3. The ChIP-qPCR for FIE-HA verified 8 of 10 FIE-HA+H3K27me3 peaks (which matches the false discovery rate predicted in Additional file 1: Table S1 B), 3 of 9 FIE-HA only peaks and showed no enrichment for the 20 regions where FIE-HA was not detected in the ChIP-seq analysis (Figure 3d). This indicates that the FIE-HA+H3K27me3 peaks are the most reliable of the FIE binding sites we identified, while the majority of the peaks identified as FIE-HA only are likely to be false positives. Therefore we focused on the FIE-HA-H3K27me3 peaks in subsequent analyses. We noted that the abundance of H3K27me3 at the H3K27me3-only sites was on average significantly lower than that for the FIE-HA+H3K27me3 sites (Figure 3e).

### FIE-HA is enriched across gene body regions

The distribution of FIE-HA and H3K27me3 was determined by plotting the ChIP enrichment of H3K27me3 and FIE-HA in a gene-centric manner. We observed the characteristic enrichment of H3K27me3 across gene bodies (Figure 3b) [16]. FIE-HA is also enriched across gene bodies (Figure 3b), but with greater enrichment at the 5′ ends in comparison to the distribution of H3K27me3. The size distributions of the FIE-HA+H3K27me3 peaks (FIE-HA peaks that overlap with H3K27me3 peaks) and all H3K27me3 peaks were compared (Figure 3c) and found to show a similar distribution at size ranges up to about 1.5 kb.

### FIE-HA genes are enriched for developmental functions

**Figure 4:**
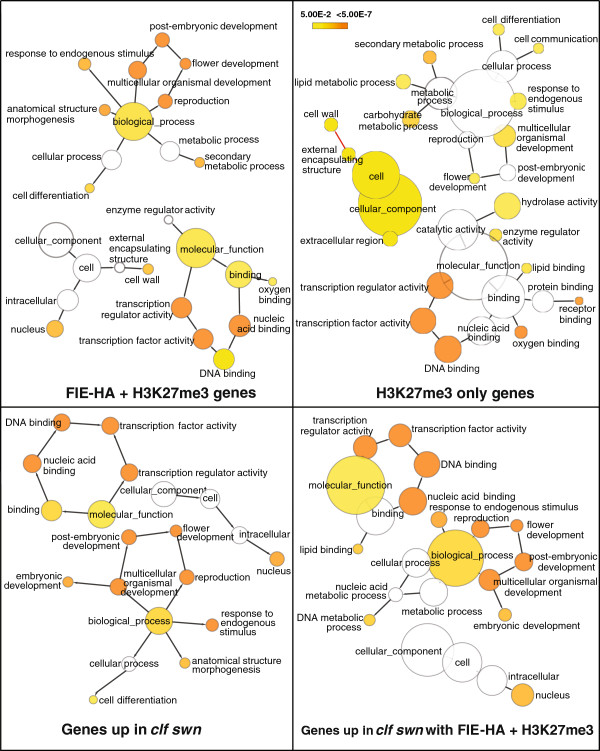
**Gene Ontology analysis of genes in the FIE-HA+H3K27me3 and H3K27me3 only datasets.** GO analysis was carried out using BinGO [45]. Enriched categories for biological process, molecular function and cellular component are shown. GOslim categories with significant enrichment in the dataset were highlighted in colour with different colours representing different levels of significance. The orange categories are most significantly overrepresented. White nodes are not significantly overrepresented. The area of a node is proportional to the number of genes in the corresponding GO category.

The genes associated with FIE-HA+H3K27me3 and H3K27me3 only peaks were used to carry out a gene ontology (GO) analysis on the two datasets (Figure 4), to determine whether there were any differences between the classes of genes with high-confidence FIE binding peaks (FIE-HA+H3K27me3) and the bulk of the H3K27me3 targets in the genome. The gene classifications that were enriched in the two datasets were similar overall, however, the GO categories for multicellular organism development, post-embryonic development, flower development and reproduction were more enriched in the FIE-HA+H3K27me3 data. In addition there were a number of individual categories that were only present in one of the datasets; most of these categories were from the H3K27me3 dataset, which has the higher number of genes.

**Figure 5:**
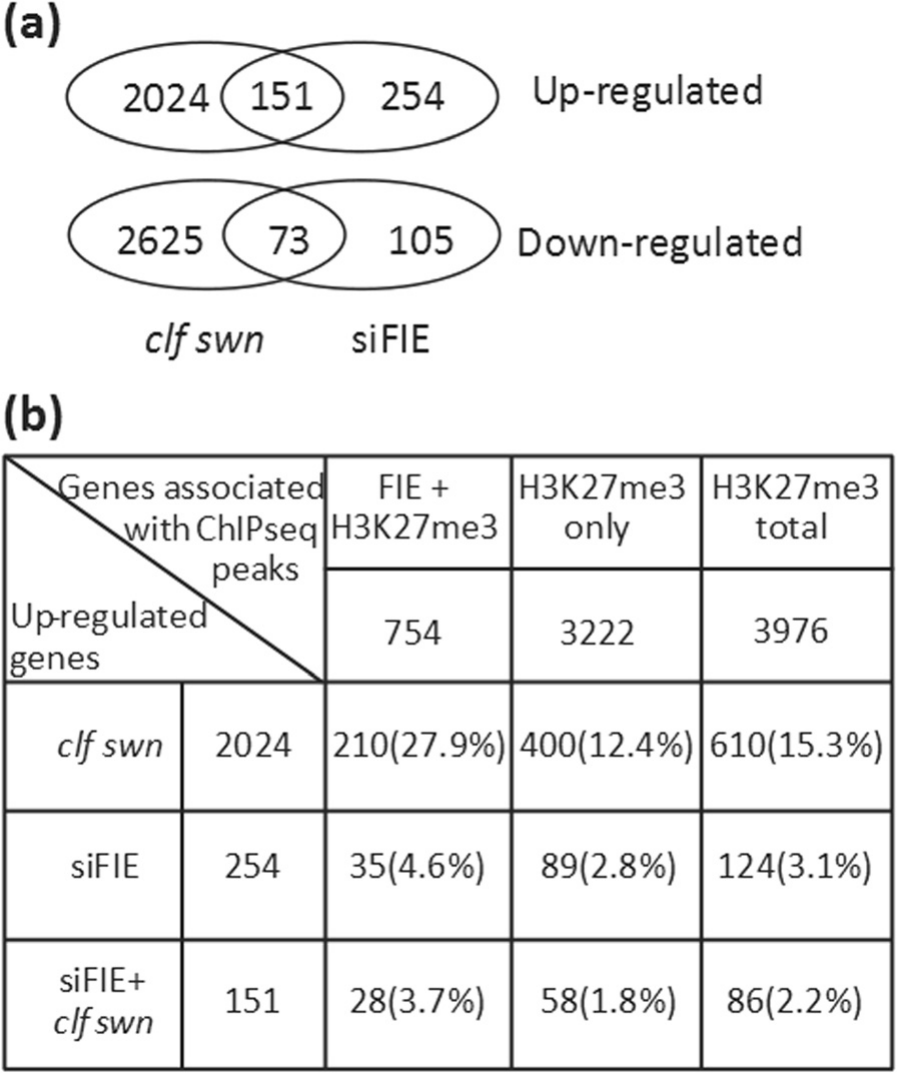
**FIE-HA+H3K27me3 genes are enriched for genes under PcG repression in vegetative tissues. (a)** Numbers of genes up- and down-regulated in *clf*-*7 swn*-*28* and siFIE seedlings compared to wildtype. Overlap of Venn diagram indicates the numbers of genes common to both datasets. **(b)** Numbers of genes up-regulated in *clf*-*7 swn*-*28*, siFIE or both that are also associated with a FIE-HA+H3K27me3 or H3K27me3 only ChIP-seq peak. Percentages are the percentage of genes associated with a ChIP-seq peak that are also in the up-regulated gene lists.

As there were differences in the functional categorisation of the genes in the FIE-HA+H3K27me3 and H3K27me3 only gene groups, we determined whether these groups of genes differed in the extent to which their expression was under PcG regulation. We analysed gene expression in seedlings of the *clf*-*7 swn*-*28* double mutant [13] and a pool of intermediate phenotype T1 plants carrying an RNAi construct against *FIE*[11] (siFIE; Additional file 5: Figure S1) using Nimblegen arrays (Figure 5; Additional file 6: Dataset S4). The results from the array experiment were verified by RT-qPCR of six genes in both *clf*-*7 swn*-*28* and siFIE compared to wildtype (Additional file 7: Figure S2). The *clf*-*7 swn*-*28* mutant shows a more severe phenotype than the siFIE plants and this is reflected in a greater number of gene expression changes in the *clf*-*7 swn*-*28* mutant (Figure 5). The primary effect expected from loss of PcG function is up-regulation of genes that are repressed by the presence of H3K27me3. However, it is clear that there are many secondary changes in gene expression, which can be seen in the large number of down- and up-regulated genes that are not associated with H3K27me3 in the *clf*-*7 swn*-*28* and siFIE expression data. The genes that are marked by H3K27me3 in wildtype plants and are up-regulated in *clf*-*7 swn*-*28* and/or siFIE include those where the presence of H3K27me3 is important in repressing gene expression; there were 610 such genes in the *clf*-*7 swn*-*28* dataset and 124 in the siFIE data with 86 genes common to both datasets (Figure 5b). We compared the frequency at which genes associated with FIE-HA+H3K27me3 or H3K27me3 were up-regulated; we found that there was about a two-fold higher proportion of the FIE-HA+H3K27me3 genes up-regulated. For example 28% of the FIE-HA+H3K27me3 genes were up-regulated in *clf*-*7 swn*-*28* compared to 12% of the H3K27me3 alone genes. Hence genes where FIE is present are more likely to be those that are primarily maintained in a repressed state by H3K27me3 and for which activators of expression are present in the seedling tissue used in these experiments. This was also reflected in the GO profiles of the genes up-regulated in *clf*-*7 swn*-*28* and siFIE which were similar to that of the FIE-HA+H3K27me3 genes (Figure 4; Additional file 8: Figures S3, Additional file 9: Figures S4, Additional file 10: Figures S5).

### High confidence FIE binding peaks contain putative GAGA Factor binding sites

**Figure 6:**
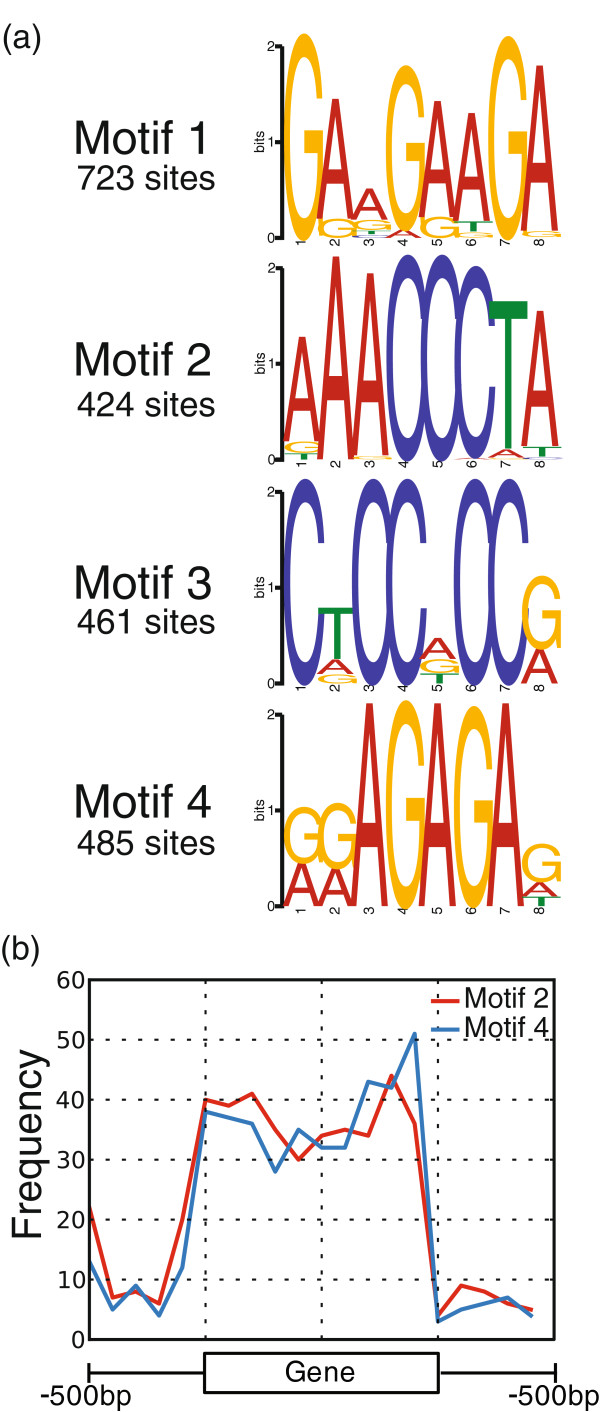
**MEME analysis of sequences in FIE-HA+H3K27me3 peaks identifies enriched sequence motifs. (a)** Motifs enriched in the FIE+H3K27me3 peaks determined by MEME analysis. **(b)** Distribution of motifs 2 and 4 with respect to gene features.

If Arabidopsis PRC2 complexes are recruited through PRE-like sequences some conserved motifs would be expected to be associated with the FIE binding peaks identified by ChIP-seq. As the FIE-HA+H3K27me3 peaks were the highest confidence FIE binding sites, we used these peak sequences in a MEME analysis. We searched for short motifs (~8 bp) on the basis that the known interaction sites of PRE-binding proteins in Drosophila are of approximately this length. This identified four motifs in the FIE-HA+H3K27me3 peaks (Figure 6a). We also carried out the same analysis on the H3K27me3 genes as well as the lower confidence FIE-HA only peaks and a control set of random promoter sequences (Additional file 11: Figure S6). Motif 1 was present in all four analyses and so was not considered further. There were no additional motifs found in the H3K27me3 only data, in the FIE-HA only data there were two additional motifs which were similar to motif 3 in the FIE-HA+H3K27me3 peaks. Comparison of the motifs in the FIE-HA+H3K27me3 peaks to motif databases using TOMTOM identified motif 2 as similar to the TBF1 binding site (telobox factor 1), motif 3 as similar to a zinc finger protein binding site and motif 4 as similar to the GAGA Factor 1/Trithorax-like binding site (Additional file 12: Figure S7). We plotted the location of the putative TBF1 and GAGA Factor binding sites relative to gene bodies and found they mirrored the location of FIE-HA+H3K27me3 peaks, being enriched across transcription units (Figure 6b).

## Discussion

We have carried out a ChIP-seq analysis to identify binding sites for the PRC2 component FIE across the Arabidopsis genome. In comparison to the numbers of H3K27me3 sites (5148) we identified fewer high confidence sites of FIE binding (723). As PRC2, and therefore FIE, is required to deposit and maintain H3K27me3, a similar numbers of peaks is expected in both experiments. The discrepancy could have some biological significance or be a technical artefact. A technical difference could be simply a consequence of the two ChIP experiments using different antibodies and targeting proteins that interact with chromatin in different ways. Histone H3 is an intrinsic part of the nucleosome structure while FIE is part of a protein complex that interacts with chromatin. Although the FIE-HA sample was cross-linked prior to immunoprecipitation, the indirect nature of the interaction between FIE and the DNA that is assayed by ChIP may make it harder to detect FIE binding regions compared to H3K27me3 regions.

Biological explanations for the low number of FIE-HA peaks compared to H3K27me3 peaks could be that there are differences in the number of PRC2 binding sites, the strength of PRC2 binding or the amount of time that PRC2 is present at a given locus. There is support for this last possibility from FRAP (Fluorescence Recovery After Photobleaching) studies in *Drosophila*[36] which suggest that polycomb complexes are not constantly bound to chromatin and that the rate of assembly of polycomb complexes differs between loci. Genome-wide comparison of H3K27me3 and PRC2 in Drosophila also identified ‘weak’ PcG sites [37] where H3K27me3 but not PRC2 was detected. The average abundance of H3K27me3 at the FIE-HA+H3K27me3 peaks was significantly higher than at the H3K27me3 only regions (Figure 3e). The genes associated with FIE-HA+H3K27me3 regions were also more likely to be up-regulated in plants that have reduced PcG function. We speculate that these genes are ones for which activators are present in vegetative tissues (with the activators regulating other genes) and hence there is a selection for increased PRC2 occupancy to maintain high levels of H3K27me3 and repression of gene expression.

A search for sequence motifs in the high confidence FIE binding sites identified four short conserved motifs. One of these was identified as being similar to the GAGA factor binding site which is a component of Drosophila PREs. The GAGA factor binds to many Drosophila PREs, but is also found in active promoters [38] and is suggested to have roles in nucleosome depletion and PcG recruitment. In plants the GAGA motif is often found within core promoter sequences [39]; however the GAGA motifs identified through FIE-HA ChIP-seq are predominantly located in gene bodies. They are not found in analyses of random sequences, indicating that there is a positive association with PRC2. The H3K27me3 only sites did not contain the GAGA motif and had lower levels of H3K27me3 than the FIE-HA+H3K27me3 peaks. The FIE-HA+H3K27me3 associated genes are also more likely to be up-regulated in plants which have a loss of PRC2 function. Based on this we speculate that the GAGA motif has a role in strengthening Polycomb recruitment to target genes for which Polycomb regulation is the primary mode of repression.

One element that functions in a PRE-like manner in plants is the RLE element in the Arabidopsis *LEC2* promoter [25]. RLE is located near a GAGA element; however this GAGA element is not required for the function of RLE. RLE is at one edge of a region of FIE-HA binding (Figure 1a), which also includes the GAGA element. The PRE-like sequence identified in the *BP* promoter does not have any associated FIE-HA binding in our high-confidence dataset, although there is evidence of FIE-HA binding across the *BP* gene body (Figure 1b). The *BP* PRE-like region has been shown to bind CLF-GFP expressed from a strong 35S promoter [27], so it may be a site of weaker PRC2 interaction. The GAGA motif has also been identified in association with LFY binding sites [40]; while some of these genes are also H3K27me3 targets, many are not. This suggests that in plants, as for Drosophila [38], the role of GAGA factor is wider than PcG function.

Although our data suggest that the GAGA factor may be a common component in the PcG regulation mechanism in plants as well as in flies, there are differences in the structures of the regions occupied by PRC2 and H3K27me3. We did not find evidence for relatively narrow regions of PRC2 binding with low H3K27me3 and depleted of nucleosomes, flanked by wide regions of H3K27me3, as seen at many Drosophila PcG targets. The observed co-localisation of FIE-HA and H3K27me3 is more reminiscent of the data in mammalian systems [41]. The association of H3K27me3 and FIE-HA binding with gene body regions appears to be particularly strong in plants compared to both mammals and insects.

## Conclusions

We have used a genome-wide ChIP-seq approach to identify FIE and hence PRC2 binding sites across the Arabidopsis genome. Based on our high-confidence dataset we find that the regions of PRC2 binding are largely within gene body regions and co-localise with H3K27me3. The emerging reports of plant PREs and our finding of GAGA motifs at FIE binding sites suggest that DNA binding proteins have a role in recruiting PRC2 in plants and that further dissection of potential PRE-like regions could help our understanding of how the PcG system is recruited to specific genes in plants.

## Methods

### Plant material

All plants were grown on MS agar plates in a 16 h light: 8 h dark photoperiod under fluorescent lights at 22°C for 12 days. Whole seedlings were harvested for ChIP experiments or RNA extraction. T1 siFIE plants (in Col ecotype) were selected on plates supplemented with kanamycin (50 mgL^-1^). The *swn*-*7 clf*-*28* mutant is sterile and was selected from the progeny of *swn*-*7 clf*-*28*/+ plants.

### ChIP-seq and bioinformatic analysis

Native chromatin immunoprecipitation (N-ChIP) was performed as described previously [42] with minor modifications. In brief, Arabidopsis seedlings were collected and ground in liquid nitrogen. Nuclei were extracted with buffers 1, 2 and 3 and chromatin was digested by MNase for 6 minutes to generate native chromatin templates consisting primarily of mononucleosomes. Native chromatin templates were incubated with anti-H3K27me3 antibody (07–449, Millipore) and antibody-bound DNA fragments were extracted. ChIP DNA fragments were sequenced by Illumina (San Diego, CA) with an Illumina Genome Analyzer (GAII) by standard procedures. A control sample of input DNA from the micrococcal nuclease digested lysates before immunoprecipitation was also sequenced.

Material for FIE-HA ChIP was cross-linked with formaldehyde and ChIP carried out as previously described [35,43] on mononucleosome sized micrococcal nuclease-digested lysates. Over 20 pull-downs were performed on sets of 1 g tissue for the FIE-HA line and the untransformed C24. ChIPs were selected for high enrichment relative to the C24 control by qPCR using a set of 6 diagnostic amplicons (Additional file 13: Table S2). DNA was pooled from the 12 immunoprecipitates that had highest enrichment and used for ChIP-seq as above except the sequencing was carried out by the Australian Genome Research Facility (Melbourne, Australia). As a control DNA extracted from the digested lysates before immunoprecipitation was also sequenced (input DNA).

The numbers of sequence reads obtained for each sample are detailed in Additional file 1: Table S1. The sequencing reads were mapped to the Arabidopsis genome (TAIR9 build) using BioKanga (http://biokanga.sourceforge.net/), allowing 2 mismatches at any position. Peaks were identified using the log_2_ ratio of signal density between two samples to determine enriched regions as candidates, followed by significance analysis on the read density from candidate peaks [44]. Briefly, each read was extended L bp (L=150 bp for H3K27me3 and L=200 bp for FIE samples respectively) from the beginning of the 5′ end to represent the fragment length. S_x_ was the normalised number of the extended reads located within a 10 bp window along a chromosome for sample x; a log_2_ ratio was then calculated on the corresponding S_x_ as log_2_R = log_2_(S_treatmen_t/S_control_). Adjacent windows that have a log_2_R above a threshold (the threshold was 3-fold enrichment when compared with input) were merged to form candidate peaks and peaks that have been separated less than 200 bp were further merged. Finally a significance test was performed using the PeakSeq algorithm [44] where a p value was obtained from binomial test on the number of reads within a candidate peak and a multiple test correction was followed to give a q value for estimation of false discovery rate. A q score of 10^-10^ was selected on the basis of maximizing the number of predicted peaks in the immunoprecipitated sample and minimizing the numbers of peaks identified in the control input DNA samples.

### GO analysis

GO enrichment analysis was performed using the BINGO 2.44 plug-in [45] in Cytoscape 2.8.3 [46] with the GOslim_plants dataset. To test for enrichment, a hypergeometric test was conducted and the Benjamini and Hochberg false discovery rate was calculated. The network of the enriched categories was presented.

### Motif analysis

MEME software (version 4.9.0) [47] was applied to yield over-represented motifs in the dataset. The width of the motif was set as 6 to 8 nucleotides. Zero or one per sequence was used for the distribution of a single motif among the sequences.

### RNA extraction and qRT-PCR

RNA was extracted from approximately 100 mg of seedlings using the RNeasy Plant Mini Kit (Qiagen) according to the manufacturer’s instructions. For quantitative RT-PCR, DNase-treated RNA was reverse transcribed using an oligo dT primer and Superscript III reverse transcriptase (Invitrogen, http://www.invitrogen.com/) and at least triplicate reactions were amplified using 7900HT Fast Real-Time PCR System (Applied Biosystems, http://www.appliedbiosystems.com/) with SYBR green. The primers used are listed in Additional file 13: Table S2. For verification of ChIP peaks qPCR was carried out using a set of genomic DNA standards that allows the comparison of values between amplicons [35].

### Expression array analysis

RNA was extracted from intermediate phenotype siFIE plants (Additional file 5: Figure S1), Col, *clf*-*7 swn*-*28* and Col using Qiagen Plant RNeasy mini kit. For each sample, three pools of 10–12 plants were used. The siFIE plants were analysed for FIE mRNA by RT-qPCR; the maximum level of FIE mRNA was found to be 10% of wildtype Col.

RNA samples were hybridised to a Roche NimbleGen Arabidopsis Gene Expression 4x72K Array (catalogue number A4511001-00-01) representing 30,361 genes, each with 2 target probes as annotated by TAIR version 6. DNAstar software was used for analysis; gene lists with higher than 2 fold de-regulation at 95% confidence compared to wild type were exported to Excel files for comparison.

## Availability of supporting data

The Nimblegen array date in this publication have been deposited in NCBI’s Gene Expression Omnibus [48] and are accessible through GEO Series accession number GSE48857 (http://www.ncbi.nlm.nih.gov/geo/query/acc.cgi?acc=GSE48857). The raw sequence data has been deposited into the NCBI Short Read Archive, accession number SRP027413.

## Abbreviations

qPCR: quantitative polymerase chain reaction.

## Competing interests

The authors declared that they have no competing interests.

## Authors’ contribution

WD, DB, WP, ED and CH participated in the design of the study, WD, DB, MR and CH carried out experiments, WD, DB, HY, JT and CH analysed data, WD and CH drafted the manuscript. All authors have read and approved the final manuscript.

## Supplementary Material

Additional file 1: Table S1 Summarises the identification of peaks from the FIE-HA and H3K27me3 datasets. Click here for file

Additional file 2: Dataset S1 Lists all H3K27me3 ChIP-seq peaks. Click here for file

Additional file 3: Dataset S2 Lists all FIE-HA ChIP-seq peaks. Click here for file

Additional file 4: Dataset S3 Lists FIE-HA peaks coincident with H3K27me3 peaks. Click here for file

Additional file 5: Figure S1 Shows phenotypes of weak, intermediate and strong T1 siFIE plants in C24. Click here for file

Additional file 6: Dataset S4 Lists of up- and down-regulated genes from microarray analysis of siFIE and *clf-7 swn-28* plants. Click here for file

Additional file 7: Figure S2 Shows RT-qPCR verification of microarray data. Click here for file

Additional file 8: Figure S3 Shows GO analysis of genes up-regulated in siFIE or *clf swn* and siFIE. Click here for file

Additional file 9: Figure S4 Shows GO analysis of genes up-regulated in siFIE or clf swn and siFIE that have FIE + H3K27me3 ChIP-seq peaks. Click here for file

Additional file 10: Figure S5 Shows GO analysis of genes up-regulated in siFIE, clf swn or both that have H3K27me3 only ChIP-seq peaks. Click here for file

Additional file 11: Figure S6 Shows additional MEME analyses to those shown in Figure 6. Click here for file

Additional file 12: Figure S7 Shows TOMTOM analyses of motifs 2 to 4. Click here for file

Additional file 13: Table S2 Lists oligonucleotide sequences used in this study. Click here for file
