# Supplementary material for: Arabidopsis Polycomb Repressive Complex 2 binding sites contain putative GAGA factor binding motifs within coding regions of genes
Source: BMC Genomics. 2013 Aug 30;14:593. doi: 10.1186/1471-2164-14-593 (PMC3766684; doi:10.1186/1471-2164-14-593)
Supplement: Additional file 5: Figure S1 — Shows phenotypes of weak, intermediate and strong T1 siFIE plants in C24. [file 1471-2164-14-593-S5.pptx]

## Slide 1
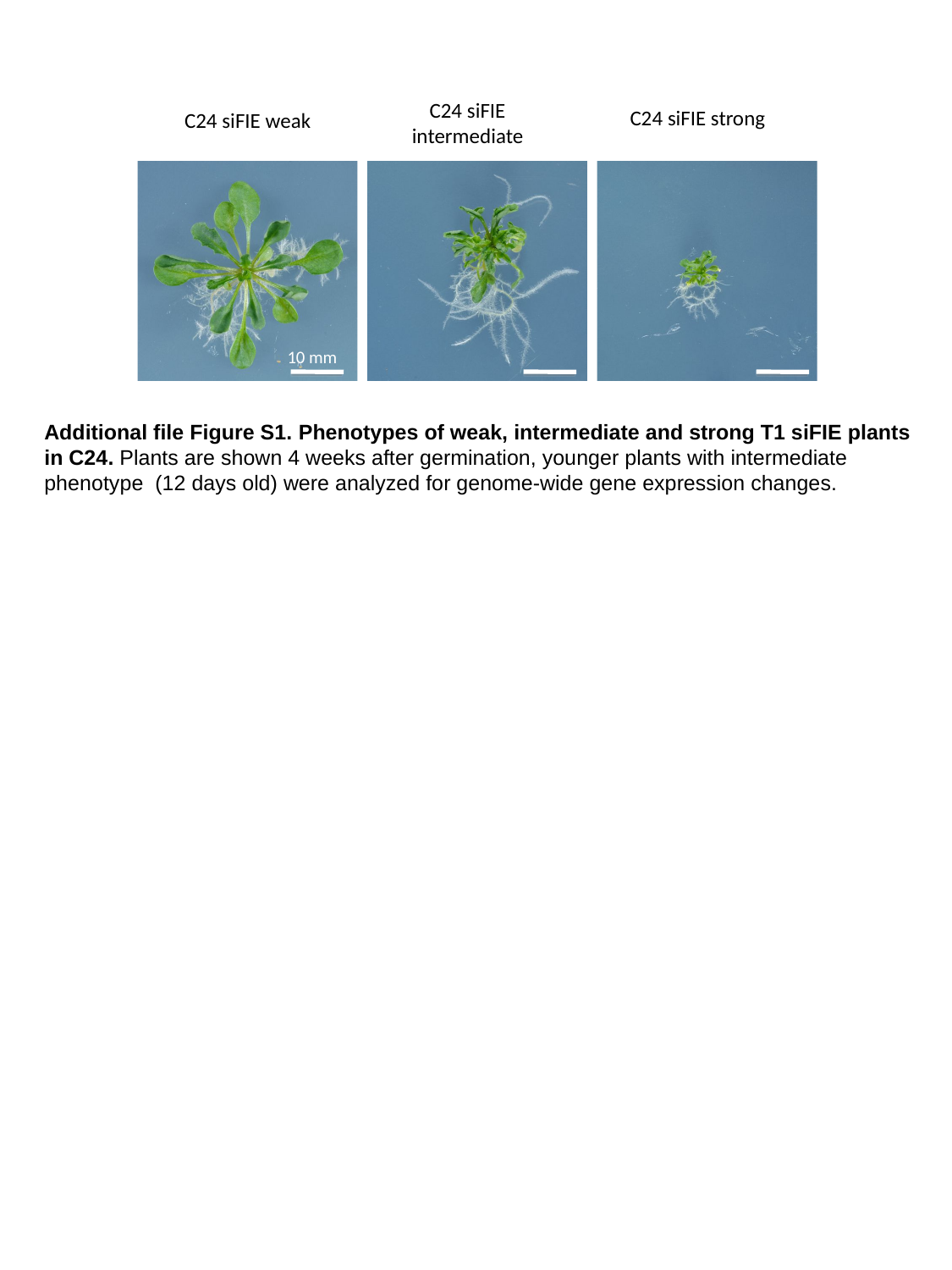

C24 siFIE intermediate
C24 siFIE strong
C24 siFIE weak
10 mm
Additional file Figure S1. Phenotypes of weak, intermediate and strong T1 siFIE plants in C24. Plants are shown 4 weeks after germination, younger plants with intermediate phenotype (12 days old) were analyzed for genome-wide gene expression changes.
siFIE(Col)
Col
