# Supplementary material for: Arabidopsis Polycomb Repressive Complex 2 binding sites contain putative GAGA factor binding motifs within coding regions of genes
Source: BMC Genomics. 2013 Aug 30;14:593. doi: 10.1186/1471-2164-14-593 (PMC3766684; doi:10.1186/1471-2164-14-593)
Supplement: Additional file 7: Figure S2 — Shows RT-qPCR verification of microarray data. [file 1471-2164-14-593-S7.pptx]

## Slide 1
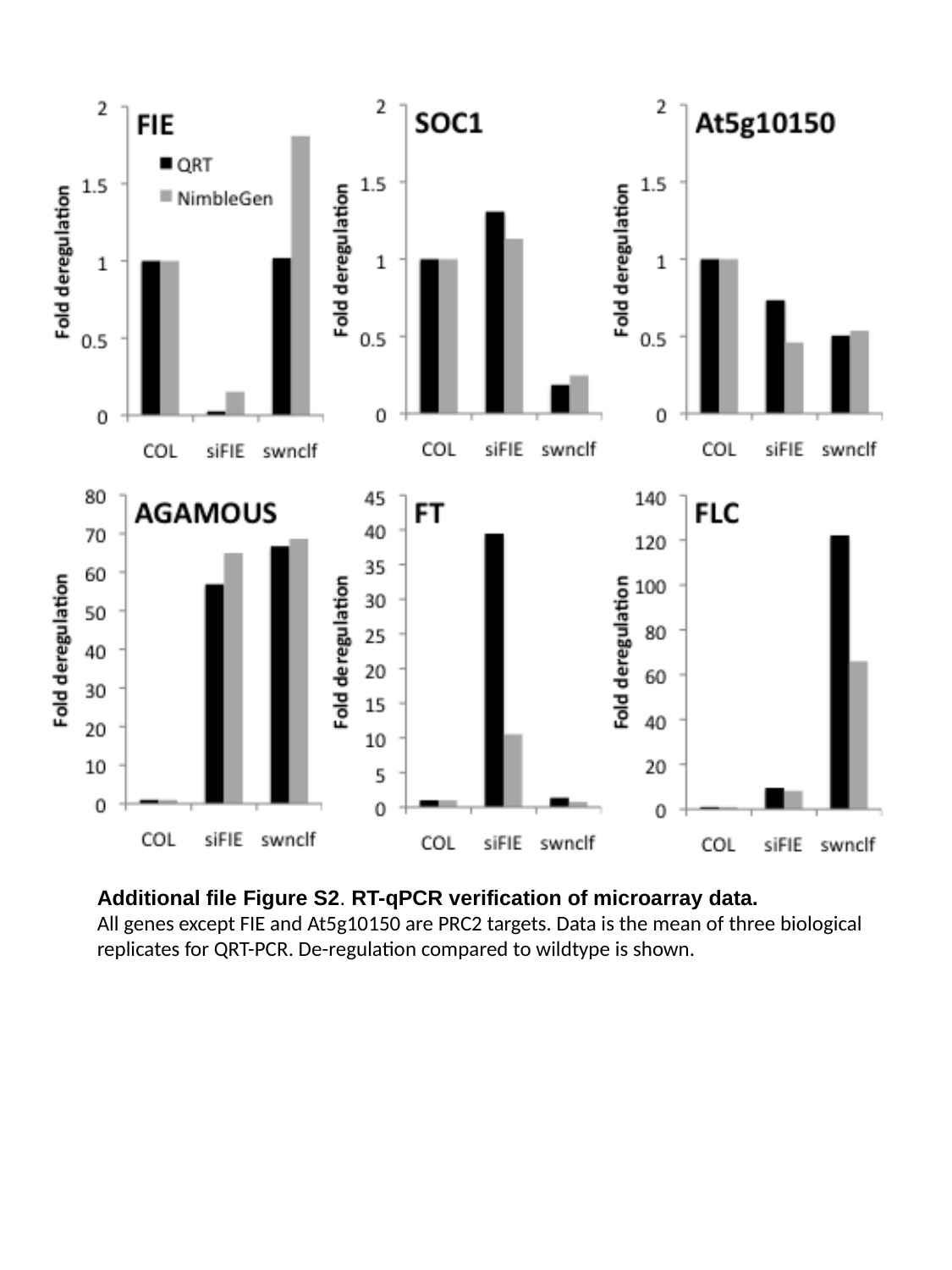

Additional file Figure S2. RT-qPCR verification of microarray data.
All genes except FIE and At5g10150 are PRC2 targets. Data is the mean of three biological replicates for QRT-PCR. De-regulation compared to wildtype is shown.
