# Supplementary figures and images for: Arabidopsis Polycomb Repressive Complex 2 binding sites contain putative GAGA factor binding motifs within coding regions of genes
Source: BMC Genomics. 2013 Aug 30;14:593. doi: 10.1186/1471-2164-14-593 (PMC3766684; doi:10.1186/1471-2164-14-593)

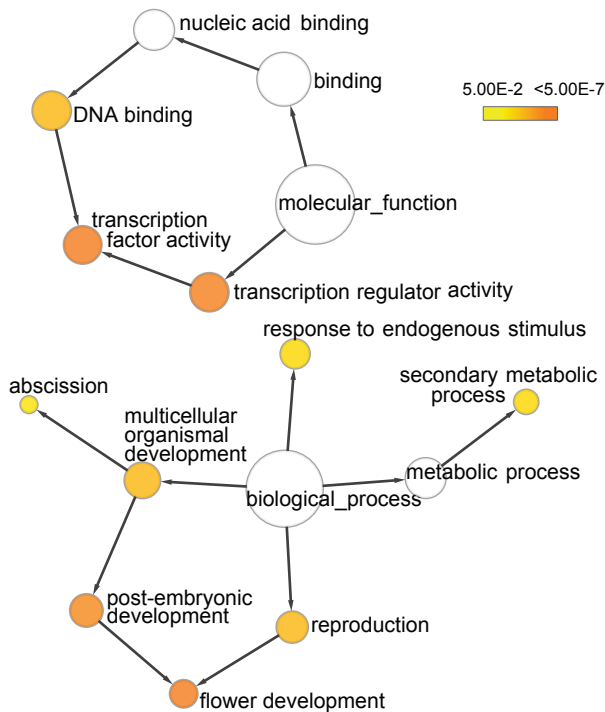

Genes up in siFIE

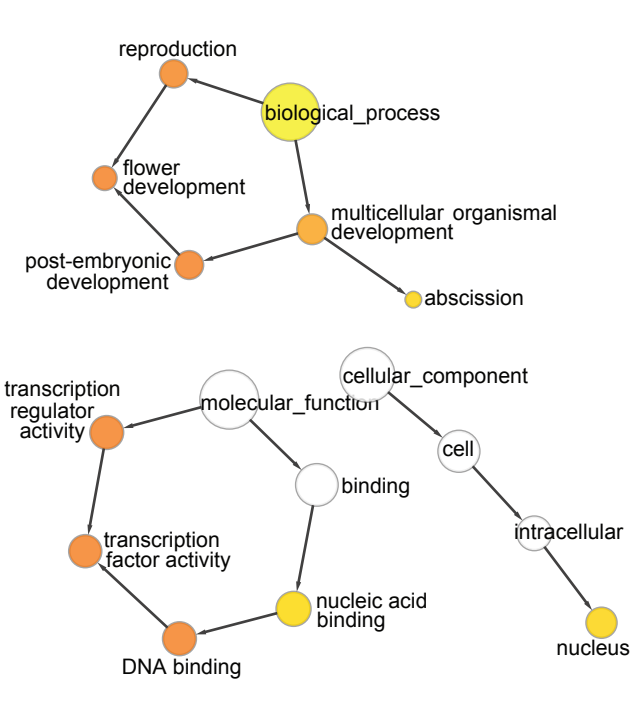

Genes up in *clfswn* and siFIE

Supplement: Additional file 8: Figure S3 — Shows GO analysis of genes up-regulated in siFIE or clf swn and siFIE. [file 1471-2164-14-593-S8.pdf]

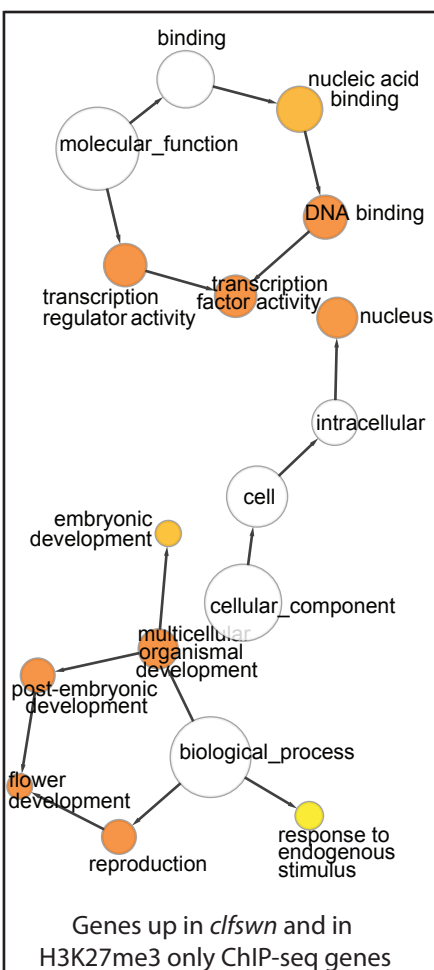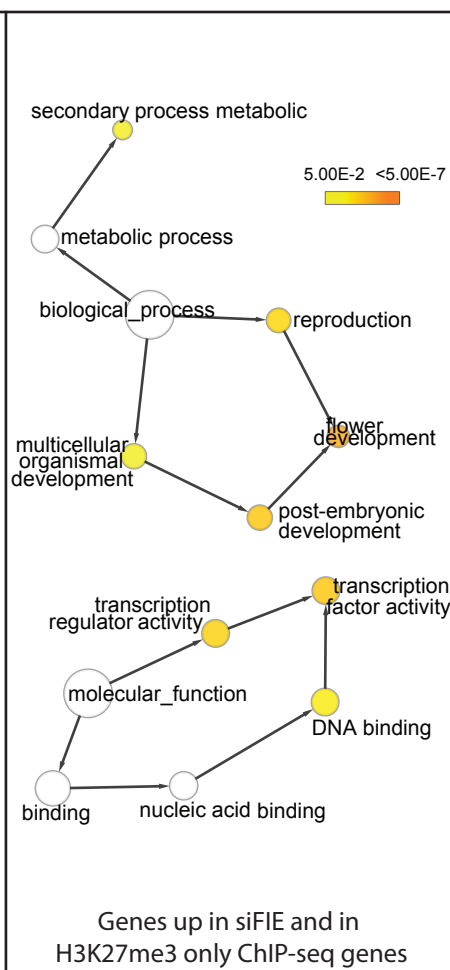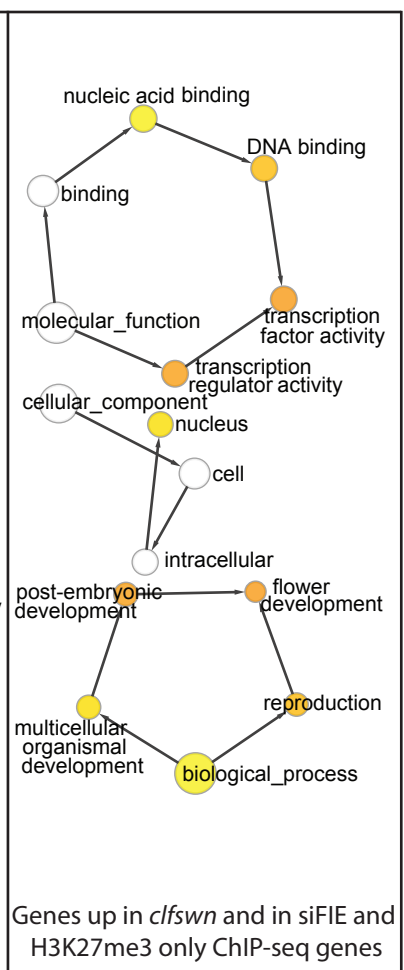

Supplement: Additional file 10: Figure S5 — Shows GO analysis of genes up-regulated in siFIE, clf swn or both that have H3K27me3 only ChIP-seq peaks. [file 1471-2164-14-593-S10.pdf]
