# Supplementary material for: Arabidopsis Polycomb Repressive Complex 2 binding sites contain putative GAGA factor binding motifs within coding regions of genes
Source: BMC Genomics. 2013 Aug 30;14:593. doi: 10.1186/1471-2164-14-593 (PMC3766684; doi:10.1186/1471-2164-14-593)
Supplement: Additional file 9: Figure S4 — Shows GO analysis of genes up-regulated in siFIE or clf swn and siFIE that have FIE + H3K27me3 ChIP-seq peaks. [file 1471-2164-14-593-S9.pdf]

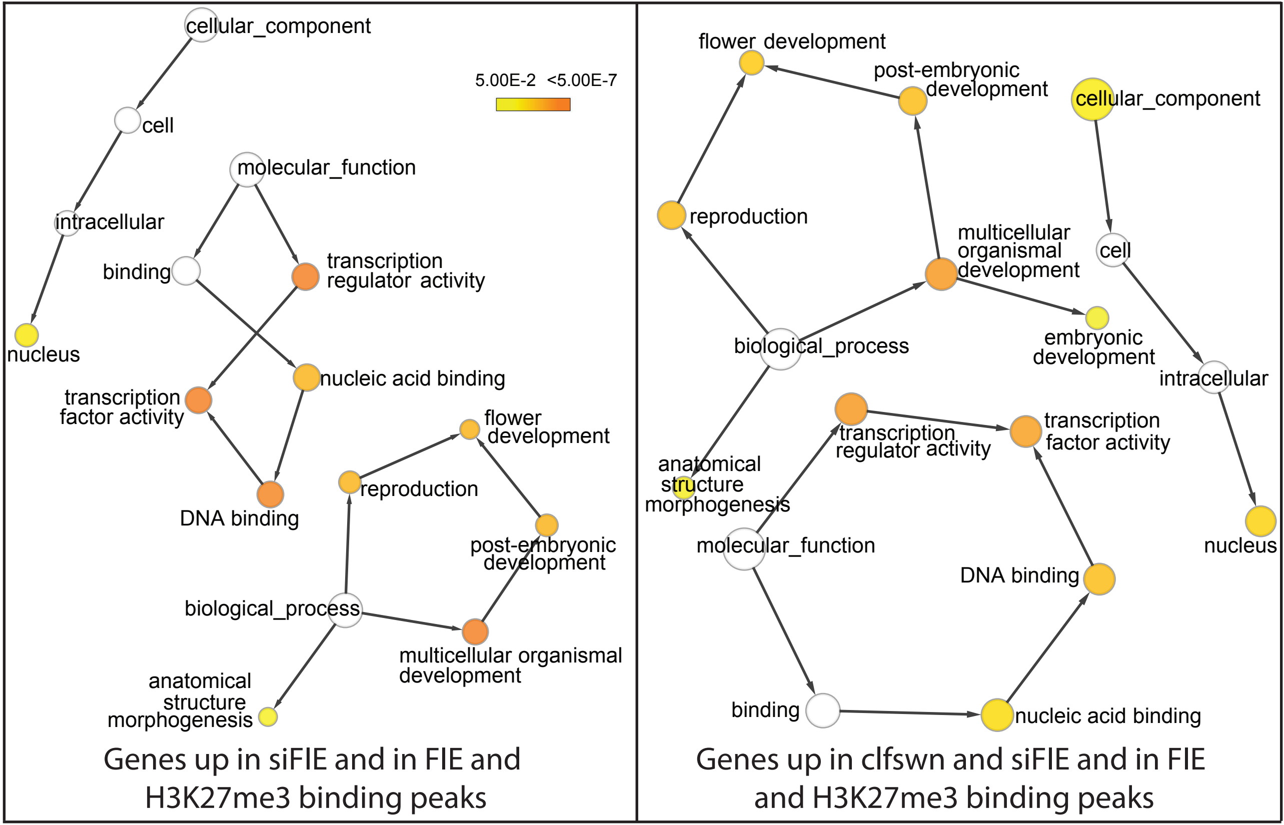
5.00E-2
<5.00E-7
cellular_component
cell
intracellular
nucleus
molecular_function
binding
transcription regulator activity
nucleic acid binding
transcription factor activity
DNA binding
flower development
reproduction
post-embryonic development
biological_process
multicellular organismal development
anatomical structure morphogenesis
Genes up in siFIE and in FIE and H3K27me3 binding peaks
flower development
post-embryonic development
cellular_component
reproduction
multicellular organismal development
cell
embryonic development
biological_process
intracellular
transcription regulator activity
transcription factor activity
anatomical structure morphogenesis
molecular_function
nucleus
DNA binding
binding
nucleic acid binding
Genes up in clfswn and siFIE and in FIE and H3K27me3 binding peaks
