# Supplementary material for: Arabidopsis Polycomb Repressive Complex 2 binding sites contain putative GAGA factor binding motifs within coding regions of genes
Source: BMC Genomics. 2013 Aug 30;14:593. doi: 10.1186/1471-2164-14-593 (PMC3766684; doi:10.1186/1471-2164-14-593)
Supplement: Additional file 11: Figure S6 — Shows additional MEME analyses to those shown in Figure 6. [file 1471-2164-14-593-S11.pdf]

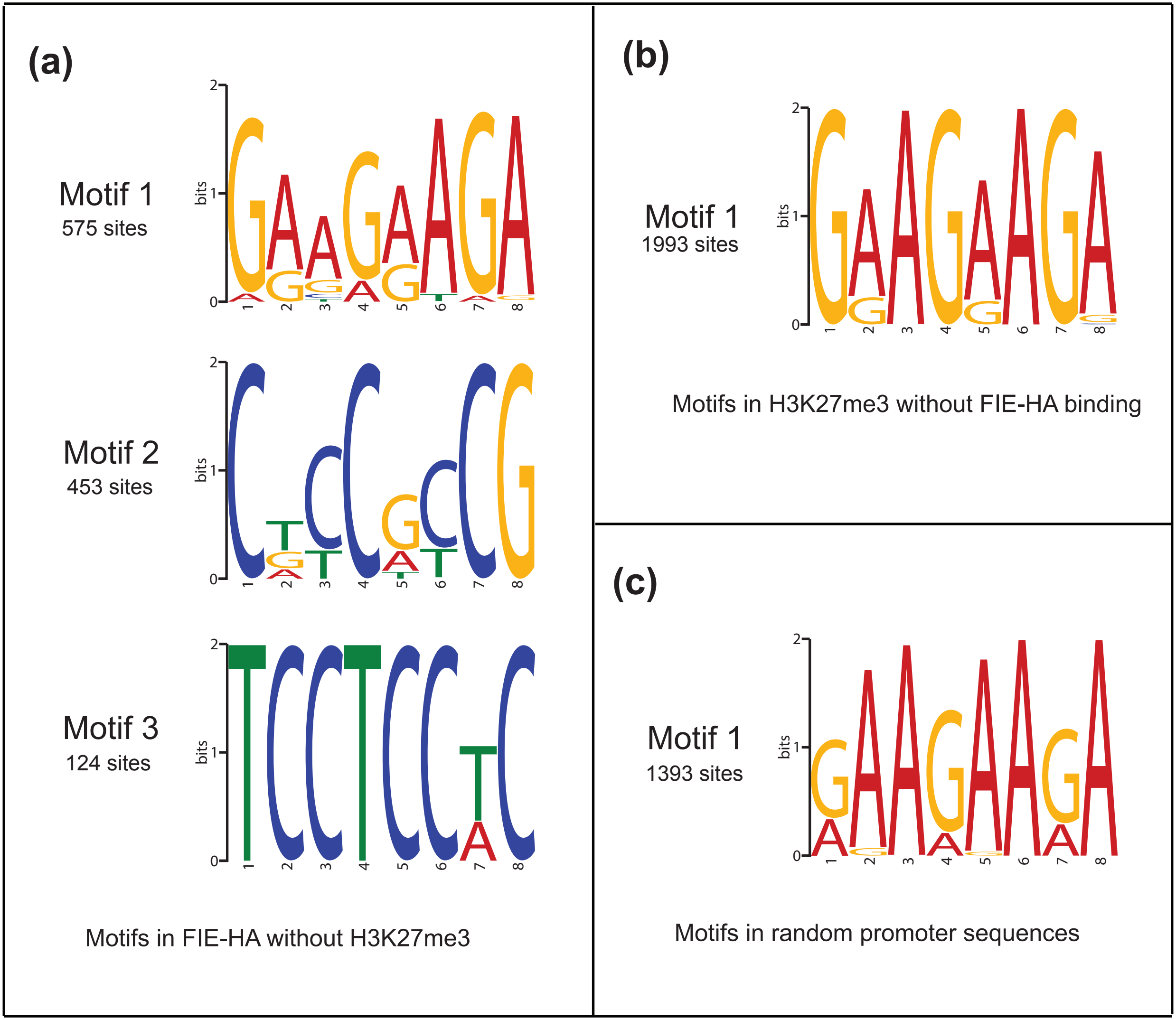
(a)
Motif 1
575 sites
Motif 2
453 sites
Motif 3
124 sites
bits
Motifs in FIE-HA without H3K27me3
(b)
Motif 1
1993 sites
bits
Motifs in H3K27me3 without FIE-HA binding
(c)
Motif 1
1393 sites
bits
Motifs in random promoter sequences
