# Supplementary material for: Arabidopsis Polycomb Repressive Complex 2 binding sites contain putative GAGA factor binding motifs within coding regions of genes
Source: BMC Genomics. 2013 Aug 30;14:593. doi: 10.1186/1471-2164-14-593 (PMC3766684; doi:10.1186/1471-2164-14-593)
Supplement: Additional file 12: Figure S7 — Shows TOMTOM analyses of motifs 2 to 4. [file 1471-2164-14-593-S12.pdf]

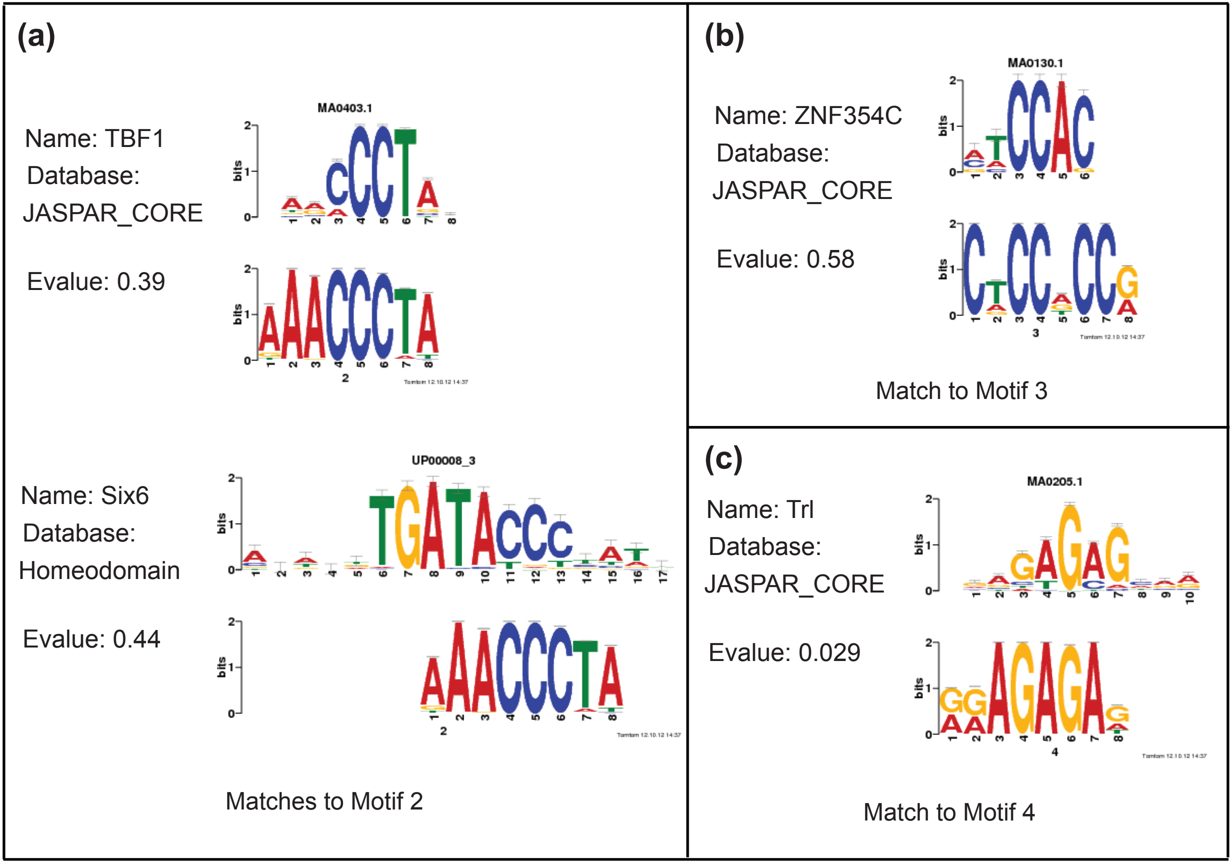

(a)
Name: TBF1
Database:
JASPAR_CORE
Evalue: 0.39
MA0403.1
bits
2
Name: Six6
Database:
Homeodomain
Evalue: 0.44
UP00008_3
bits
2
Matches to Motif 2
(b)
Name: ZNF354C
Database:
JASPAR_CORE
Evalue: 0.58
MA0130.1
bits
3
Match to Motif 3
(c)
Name: Trl
Database:
JASPAR_CORE
Evalue: 0.029
MA0205.1
bits
4
Match to Motif 4
